# Hypothalamic and amygdalar cell lines differ markedly in mitochondrial rather than nuclear encoded gene expression

**DOI:** 10.1186/1471-2164-14-413

**Published:** 2013-06-21

**Authors:** Dhwanil A Dalwadi, Rosalie M Uht

**Affiliations:** 1 Department of Pharmacology and Neuroscience, University of North Texas Health Science Center, 3500 Camp Bowie Blvd, Fort Worth, TX 76107, USA; 2 Institute for Aging and Alzheimer’s Research, University of North Texas Health Science Center, 3500 Camp Bowie Blvd, Fort Worth, TX 76107, USA

**Keywords:** Stress, Hypothalamus, Amygdala, Mitochondria, Oxidative phosphorylation, NADH dehydrogenase, Cytochrome c oxidase, Cytochrome b, Mitochondrial genes, Glucocorticoids

## Abstract

**Background:**

Corticotropin-releasing hormone (CRH) plays an important role in regulating the mammalian stress response. Two of the most extensively studied neuronal populations that express CRH are in the hypothalamus and amygdala. Both regions are involved in the stress response, but the amygdala is also involved in mediating response to fear and anxiety. Given that both hypothalamus and amygdala have overlapping functions, but their CRH-expressing neurons may respond differently to a given perturbation, we sought to identify differentially expressed genes between two neuronal cell types, amygdalar AR-5 and hypothalamic IVB cells. Thus, we performed a microarray analysis. Our hypothesis was that we would identify differentially expressed transcription factors, coregulators and chromatin-modifying enzymes.

**Results:**

A total of 31,042 genes were analyzed, 10,572 of which were consistently expressed in both cell lines at a 95% confidence level. Of the 10,572 genes, 2,320 genes in AR-5 were expressed at ≥ 2-fold relative to IVBs, 1,104 genes were expressed at ≥2-fold in IVB relative to AR-5 and 7,148 genes were expressed at similar levels between the two cell lines. The greatest difference was in six mitochondrial DNA-encoded genes, which were highly abundant in AR-5 relative to IVB cells. The relative abundance of these genes ranged from 413 to 885-fold according to the microarray results. Differential expression of these genes was verified by RTqPCR. The differentially expressed mitochondrial genes were cytochrome b (MT-CYB), cytochrome c oxidase subunit 1 and 2 (MT-CO1 and MT-CO2) and NADH-ubiquinone oxidoreductase chain 1, 2, and 3 (MT-ND1, MT-ND2, MT-ND3).

**Conclusion:**

As expected, the array revealed differential expression of transcription factors and coregulators; however the greatest difference between the two cell lines was in genes encoded by the mitochondrial genome. These genes were abundant in AR-5 relative to IVBs. At present, the reason for the marked difference is unclear. The cells may differ in mtDNA copy number, number of mitochondria, or regulation of the mitochondrial genome. The specific functions served by having such different levels of mitochondrial expression have not been determined. It is possible that the greater expression of the mitochondrial genes in the amygdalar cells reflects higher energy requirements than in the hypothalamic cell line.

## Background

Any change that results in homeostatic imbalance due to exogenous or endogenous stimuli may be considered a stressor. This, in turn, initiates a complex signaling cascade of stress-inducible proteins and transcription factors that serves to return the cell to homeostatic conditions. The appropriate cellular response is determined by the ability to successfully meet the physiological demands of stress, and is paramount for survival. This is a multi-system process, and in mammals, activation of the hypothalamic-pituitary-adrenal (HPA) axis plays a key role.

The stress-response can be divided into three categories: behavioral, autonomic and hormonal [1]. The behavioral response includes the skeletal motor response to a stressor, whereas the autonomic response, mediated though the sympathetic nervous system, increases activity of organs, such as the heart and lungs, for fight or flight responses [1]. The hormonal response provides fuel for such activities. The neuropeptide corticotropin-releasing hormone (CRH) is believed to be involved in all three stress-responses, involving different brain regions [1]. The behavioral response is initiated in part by the amygdala, which contains CRH-expressing neurons and mediates fear-associated behavior such as fight or flight. CRH neurons in the amygdala and paraventricular nucleus project to the locus coeruleus, which in turn sends descending fibers to the brainstem, which is responsible for autonomic responses ([2,3] and references therein). The hormonal response is initiated by activation of the HPA axis. This activation leads to the release of CRH from the paraventricular nucleus of the hypothalamus (PVH) into the portal circulation of the median eminence. In contrast, the amygdalar CRH system is more sensitive to psychological stressor than the PVH CRH system given that psychological stress increases CRH levels in the amygdala but not in the PVH [4]. However, CRH increase in the amygdala does not elicit a hormonal response and may be contributing to psychological stress-evoked behavior such as hyperarousal [4]. Due to the wide range of effects of CRH, its expression needs to be tightly regulated, and its dysregulation is associated with profound neuropsychiatric consequences, in particular, mood disorders such as depression and anxiety [5].

In this study, we utilized hypothalamic (IVB) and amygdalar (AR-5) rat clonal cell lines. Particularly for such a heterogeneous tissue as the CNS, *in vitro* systems are invaluable for studying signaling mechanisms and gene regulation [6]. The hypothalamic IVB cell line is an immortalized rat fetal hypothalamic cell line developed from rat-primary hypothalamic culture by retroviral transformation [7]. These cells express CRH mRNA, exhibit CRH immunoreactivity, and co-express vasopressin and Type-1 CRH receptors, suggesting a parvocellular phenotype [7]. The amygdalar AR-5 cell line was prepared the same way as the IVBs and is similar to primary amygdalar cell culture in response to known regulators of amygdalar CRH [8].

The aim of this study was to identify differentially expressed genes in the two cell types at basal levels. Our hypothesis was that the most significant difference between the two lines would be a differential expression of transcription factors, coregulators and/or chromatin-modification enzymes.

## Methods

### Cell culture and treatment

Rat AR-5 amygdalar and IVB hypothalamic cell lines were used in this experiment. AR-5 and IVB cells were cultured in phenol red-free DMEM/F12 media (Hyclone) supplemented with 10% newborn calf serum (NCS) (Gemini Bioproducts), 2 mM L-glutamine, 1 mM sodium pyruvate, 0.1 mM nonessential amino acids, and 100 U/mL penicillin/streptomycin (all from Cellgro, Mediatech Inc.).

For the microarray experiment, 10^5^ cells were plated on Nunc 6-well plates (Nalge Nunc International). After 24 hours, cells were washed with PBS (137 mM NaCl, 2.7 mM KCl, 4.3 mM Na_2_HPO_4_ and 1.47 mM KH_2_PO_4_; pH 7.4) and maintained in media containing charcoal-stripped NCS for 48 hours.

### RNA isolation

Total RNA was extracted using Tri-reagent® (MRC Inc.) according to manufacturer’s protocol. Briefly, cells were suspended in 1 mL of Tri-reagent and incubated at room temperature for 5 minutes. Subsequently, 0.2 mL of chloroform was added and the homogenate was shaken vigorously for 15 seconds and incubated at room temperature for 10 minutes. Samples were centrifuged at 12,000 g for 15 minutes at 4°C. The aqueous phase was then combined with equal volume of isopropanol, incubated at room temperature for 10 minutes and centrifuged at 12,000 g for 15 minutes at 4°C. The resulting RNA pellet was washed with 1 mL of 75% ethanol, centrifuged (at 7500 g, 5 min, 4°C), air-dried for 10 minutes and dissolved in 20 μL of nuclease-free water (Sigma). The RNA concentration was measured using a Bio-tek plate reader, and RNA integrity was assessed using an Agilent2100 RNA nano-chip.

### Gene expression analysis

Sample labeling, hybridization and data extraction was performed by the UTSW microarray core facility; the Affymetrix rat genome 230 2.0 array was used. ArrayStar (DNASTAR, Inc.) was used to obtain the gene list and compare the relative gene expression between the AR-5 and IVB cell lines. The experiment was performed in triplicate, and genes that were consistently expressed in the two cell lines at a 95% confidence level were analyzed further. A hierarchical clustering method was used to identify functional groups of genes that are abundant in each cell line.

Measure of differential expression was calculated by setting the IVB expression levels as baseline and is expressed as fold difference. Genes expressed at 2-fold or greater were considered to be expressed at a higher level in the AR-5 and genes expressed at 0.5 fold or less were considered to be abundant in the IVBs.

The gene expression data was combined with the information from the Gene Ontology (GO) database, using the Database for Annotation, Visualization and Integrated Discovery (DAVID) bioinformatics database [9,10]. DAVID is a functional annotation tool that allows the investigator to assign empirically determined biological functions to a large set of gene list. The GO database (a tool within DAVID) was used to identify biological processes associated with the genes in the 95% confidence level that met the 2-fold cutoff criteria. To search for reported physical and/or functional associations between the enriched protein coding genes, the data was analyzed using the Search Tool for the Retrieval of Interacting Genes/Proteins (STRING) database [11].

### Validation of microarray results by RTqPCR

Complementary DNA (cDNA) was synthesized using the Verson cDNA kit (Thermo Fisher Scientific), following the manufacturer’s protocol with the following modifications. A 1 μg sample of total RNA was used as the template, reverse gene specific primers were used and cDNA was synthesized at 47.8°C for 1 hour. RTqPCR was performed using the BIORAD thermocycler to compare the expression levels of MT-CO1, MT-CO2, MT-CYB, MT-ND1, MT-ND2 and MT-ND3 between the two cell lines; 60S ribosomal protein L27a (RpL27a) was used as the reference gene. Primers used for each gene can be found in Table 1.

**Table 1:** List of primers used to verify the expression of 6 mitochondrial genes identified in the array

MT-CO1_F	TCACTGCCAGTATTAGCAGCAGGT
MT-CO1_R	TCTGGGTGGCCGAAGAATCAGAAT
MT-CO2_F	ACACACACAAGCACAATAGACGCC
MT-CO2_R	AATTCGTAGGGAGGGAAGGGCAAT
MT-CYB_F	ACATTCCGCCCAATCACCCAAATC
MT-CYB_R	TACTGGTTGGCCTCCGATTCATGT
MT-ND1_F	AAGCGGCTCCTTCTCCCTACAAAT
MT-ND1_R	GAAGGGAGCTCGATTTGTTTCTGC
MT-ND2_F	ACTACCCGAAGTCACCCAAGGAAT
MT-ND2_R	CAGGCGCCAACAAAGACTGATGAA
MT-ND3_F	TGAATGTGGCTTCGACCCAACAAG
MT-ND3_R	TTGTTTGAATCGCTCATGGGAGGG
RpL27a_F	TGTAGGCTCCATCCAGCTTCACTT
RpL27a_R	TCAGTTTGCAGTGCTGATGTGCTG

The cycling parameters were: initial melting step at 95°C for 15 sec, and amplification at 95°C for 5 sec, then 60°C for 30 sec. The amplification steps were repeated for a total of 40 cycles.

The ΔΔC(t) method was used to obtain the fold difference. The C(t) values for IVBs were used as the reference value and the data was normalized to RpL27a.

### Statistical analysis

Three biological replicates were analyzed for each cell line. A statistical cutoff of 5% False Discovery Rate (FDR) was generated with a moderate t-test with Benjamini Hochberg multiple testing correction. A 2-fold cutoff was also applied to identify differentially expressed genes.

For the RTqPCR data, the data represents the average of 11 biological replicates, the statistical significance was determined by performing one-tailed student’s t-test and p ≤ 0.05 was considered significant.

To measure gene-enrichment in biological processes, Expression Analysis Systematic Explorer (EASE) score was used, which is a modified Fisher Exact p-value. An EASE score of < 0.05 was used to identify significantly enriched GO terms.

## Results and discussion

### Comparison between AR-5 and IVB gene expression patterns

**Figure 1:**
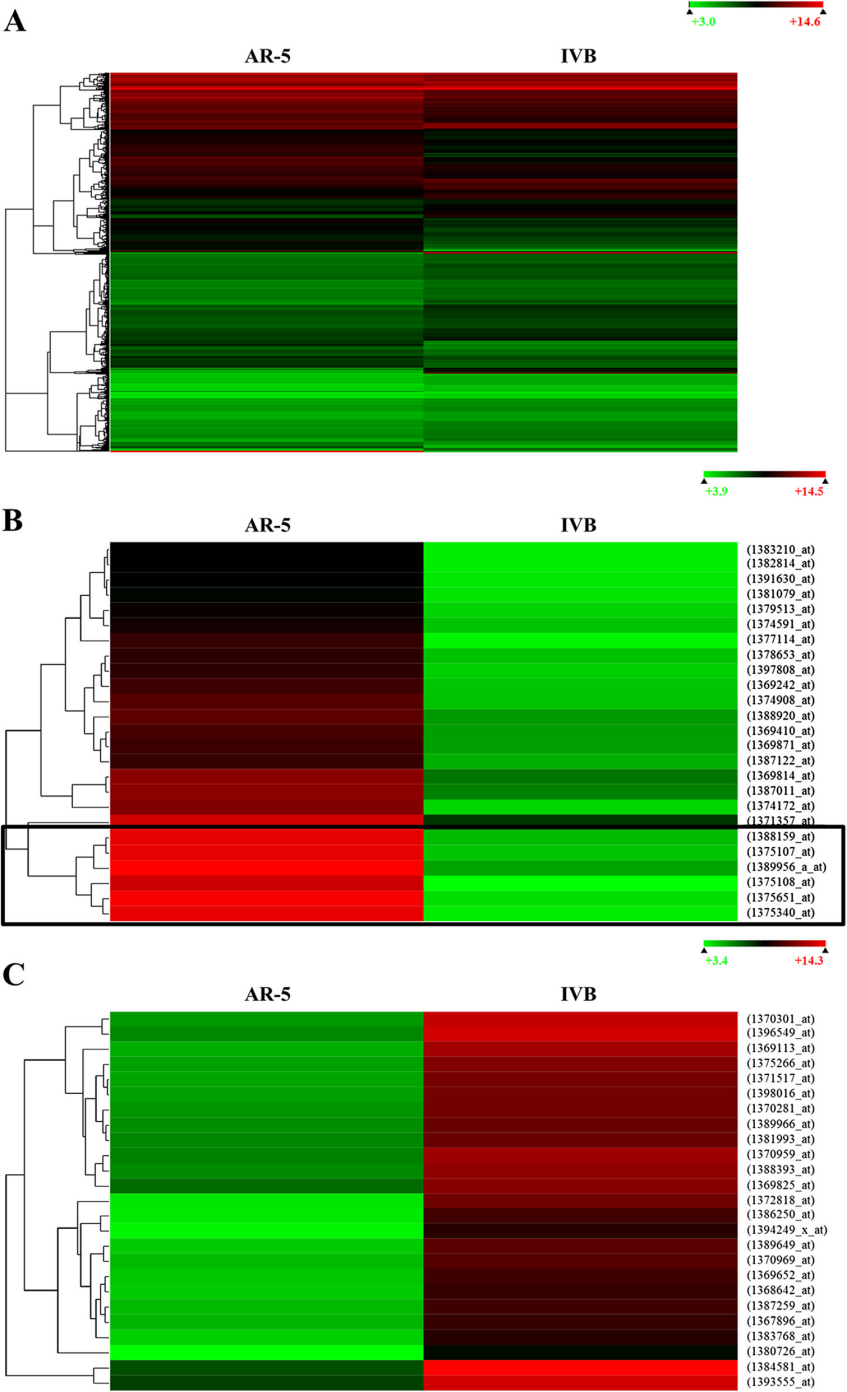
**Hierarchal clusters showing the relative abundance of genes in AR**-**5 cells as compared to abundance of genes in IVB cells (Red = high expression, Green = low expression).** (**A**) Expression of 10,572 genes that were consistently expressed in the two cell lines at a 95% confidence level from 3 biological replicates. (**B**) A subcluster of genes that are highly expressed in AR-5, relative to IVBs. The cluster outlined in the box represents the mitochondrial DNA-encoded genes: MT-CO1, MT-CO2, MT-CYB, MT-ND1, MT-ND2 and MT-ND3. (**C**) Subcluster of genes highly expressed in IVBs relative to AR-5 s.

An Affymetrix microarray platform was used to obtain a gene expression profile of AR-5 and IVB cell lines. As explained previously, the expression levels of AR-5 and IVB were compared, and genes with a fold difference of ≥ 2 were considered relatively abundant in AR-5, and genes with a fold difference of ≤ 0.5 were considered abundant in IVB. A total of 31,042 genes were analyzed on the array, out of which 10,572 genes were consistently expressed in the two cell lines at a 95% confidence level. Out of the 10,572 genes, 2,320 genes were expressed at ≥2-fold, relative to expression in IVBs; 1,104 genes were expressed at ≥2-fold, relative to expression in AR-5 and 7,148 genes were expressed at similar levels in the two cell lines. Figure 1A shows the hierarchical clustering of all the genes in the 95% confidence level, where red indicates highly expressed genes and green indicates low expression of genes.

Out of the 2,320 genes that were comparatively abundant in AR-5s, 6 genes had a fold difference of >410 (Figure 1B). These are Cytochrome b (MT-CYB), Cytochrome c oxidase subunit 1 (MT-CO1), Cytochrome c oxidase subunit 2, (MT-CO2), NADH-ubiquinone oxidoreductase chain 1, 2 and 3 (MT-ND1, 2 and 3, respectively), all of which are encoded by the mitochondrial genome. Out of the 1,104 genes that were comparatively abundant in IVBs, 6 genes had a fold difference of > 80 (Figure 1C). These genes are sorting nexin 12 (Snx12), gremlin 1 (Grem1), actin gamma 1 (Actg1), collectin sub-family member 12 (Colec12), eukaryotic translation elongation factor 2 (Eef2) and matrix metallopeptidase 2 (Mmp2). We focused on the 6 mitochondrial genes because there was a >410 fold difference in mitochondrial genes between the two cell lines.

**Figure 2:**
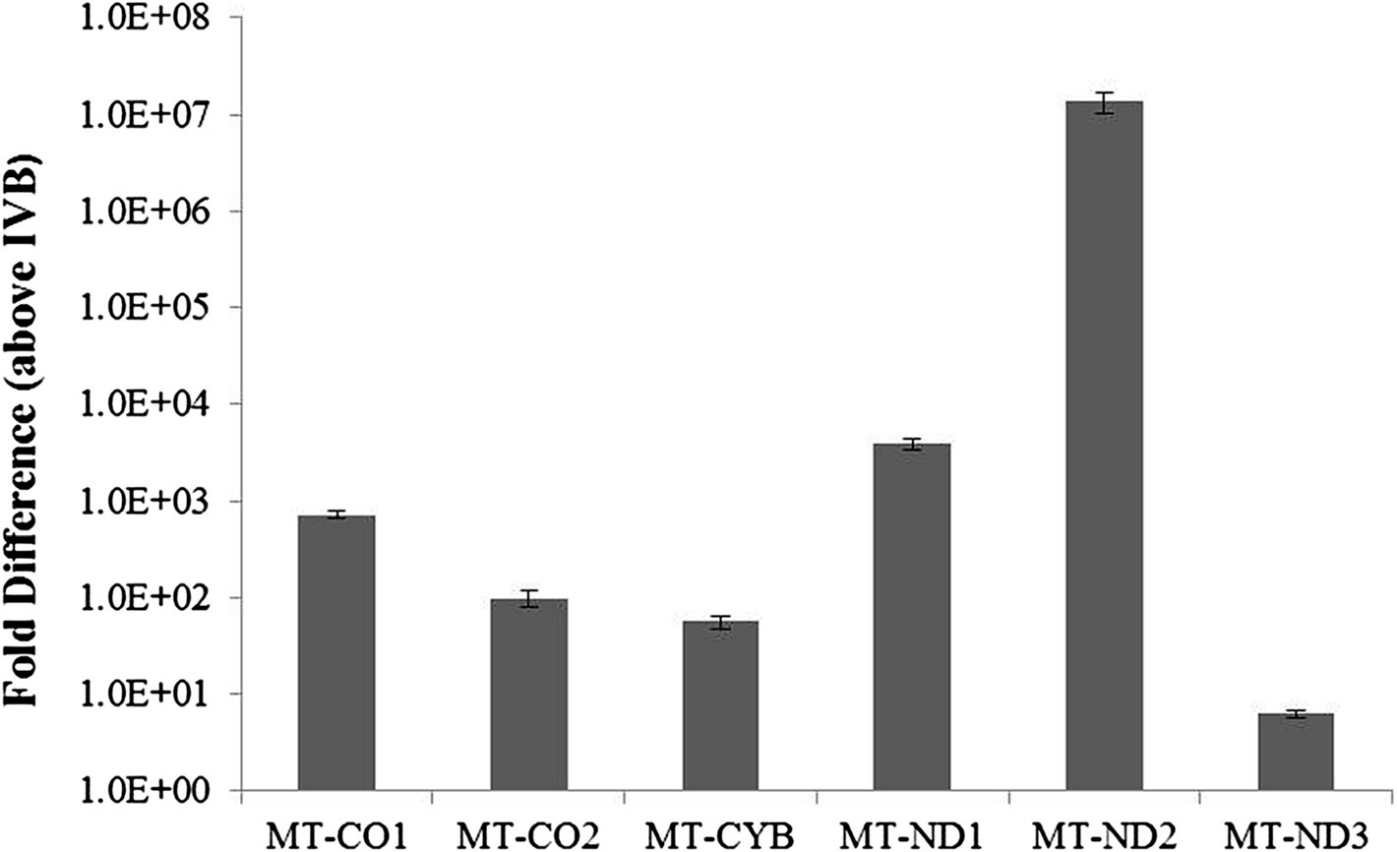
**Verification of selected genes from the microarray data by RTqPCR.** The six mitochondrial genes that were expressed at 400-fold or greater in the AR-5 line as compared to the IVB line, were selected for verification. The ΔΔC_T_ method was used for quantitation and the data was normalized to 60S ribosomal protein L27a (RpL27a). All p-values are ≤ 0.0005. The genes verified by RTqPCR were (MT-CO1) Cytochrome c oxidase subunit 1, (MT-CO2) Cytochrome c oxidase subunit 2, (MT-CYB) Cytochrome b, (MT-ND1) NADH ubiquinone oxidoreductase chain 1, (MT-ND2) NADH ubiquinone oxidoreductase chain 2, and (MT-ND3) NADH ubiquinone oxidoreductase chain 3.

To validate the microarray results, the relative expression of the 6 mitochondrial genes was analyzed by RTqPCR. The data was normalized to RpL27a, and the results shown in Figure 2 confirm the finding of the microarray results. The greatest difference in expression is evident for MT-ND2, which is expressed at 10^7^-fold greater in AR-5, relative to the IVB cell line.

Mitochondria play a crucial role in cellular physiology and are among the first organelles to respond to various stressors that might influence cell homeostasis. Adaptive stress response involves changes in mitochondrial functions, which enable them to adjust thermogenesis, bioenergetics, oxidative and apoptotic responses [12]. The HPA axis plays a central role in the neuroendocrine response to stress, thus it was surprising to see such marked differences in the expression of oxidative phosphorylation (OXPHOS) genes in the amygdalar cell line rather than in a cell line representative of the neuroendocrine component of the HPA axis (PVH). Cell-specific variation in gene expression is expected but a 400-fold or greater difference is very unusual.

It has been established that mitochondrial density differs between tissues [13]. Thus, it is not surprising that a high energy-consuming organ like the brain has a high mitochondrial density. When regional variation in mouse brain was assessed in 39 brain regions of adult mice, no significant difference was detected between the amygdala and hypothalamus, even though there were differences between other regions like the ventral tegmental area and cerebellum [14]. Several reasons may be offered for the discrepancy between the latter finding and our data, such as an *in vitro* vs. *in vivo* settings and species differences of mice *vs*. rats. In addition, our two leading theories are, first, that in the rat brain there is a difference in mitochondrial DNA copy number and/or the total number of mitochondria between the amygdala and the hypothalamus, and second, that there is differential regulation of mitochondrial genes. We will be testing these theories.

The mitochondrial genome consists of ~16 kb and codes for 37 genes. Of these, 13 are protein-coding genes and form subunits of the electron transport chain (ETC). The ETC consists of 5 complexes and a total of 97 genes make up the ETC, of which 84 are encoded by the nuclear genome. The expression of the nuclear-encoded mitochondrial genes did not differ between the two cell lines, as per our criteria for differential expression (Additional file 1: Table S1, category “Electron Transport Chain”). This underscores that mitochondria are signal contributors to the differences between the two cell lines.

### Gene ontology

**Figure 3:**
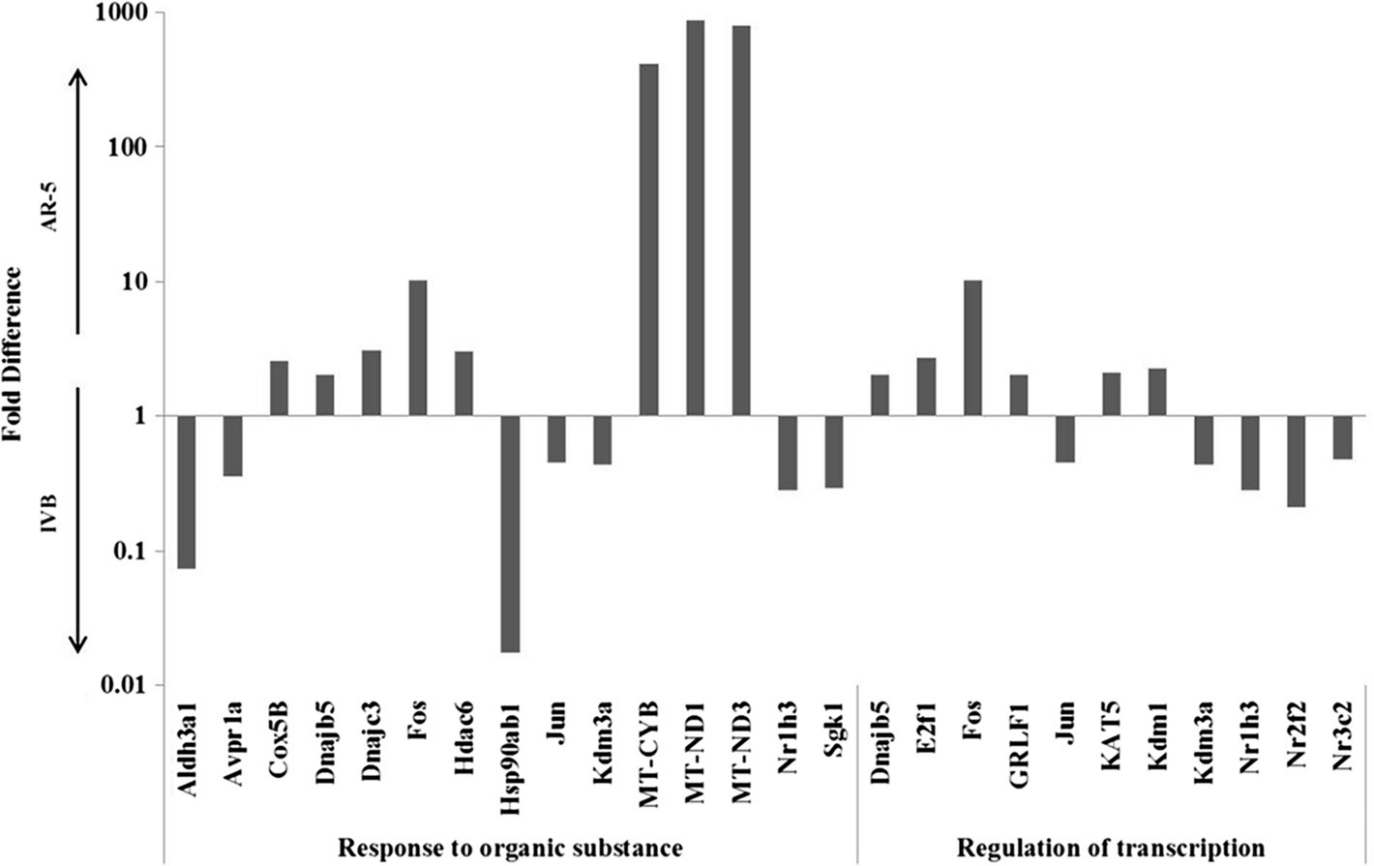
**Genes expressed at** ≥**2**-**fold (as per microarray) in each cell line, relative to each other, were analyzed using the DAVID bioinformatics tool.** The graph shows an example of two biological processes (GO Terms) that have an EASE score < 0.05: “response to organic stimuli” and “regulation of transcription”. A fold-difference > 1 indicates high expression in AR-5 and < 1 indicates high expression in IVB cells. The following genes are shown in the graph: (Aldh3a1) aldehyde dehydrogenase 3A1, (Avpr1a) Arginine vasopressin receptor 1A, (Cox5B) cytochrome c oxidase subunit Vb, (MT-CYB) cytochrome b, (Dnajb5) DnaJ (Hsp40) homolog subfamily B member 5, (Dnajc3) DnaJ (Hsp40) homolog subfamily C member 3, (E2f1) E2F transcription factor 1, (Fos) FBJ osteosarcoma oncogene, (GRLF1) glucocorticoid receptor DNA binding factor 1, (Hdac6) histone deacetlyase 6, (Hsp90ab1) heat shock protein 90 kDa alpha class B member 1, (Jun) Jun proto-oncogene, (KAT5) Lysine (K) acetyltransferase 5, (Kdm1) Lysine (K)-specific demethylase 1, (kdm3a) lysine (K)-specific demethylase 3A, (MT-ND1-3) NADH-ubiquinone oxidoreductase chain 1–3, (Nr1h3) liver x receptor alpha, (Nr2f2) COUP transcription factor 2, (Nr3c2) mineralocorticoid receptor, (sgk1) serum/glucocorticoid regulated kinase 1.

To identify the biological processes associated with the genes from the microarray, differentially expressed genes were interrogated using the DAVID program. A total of 235 biological processes were enriched in AR-5s, 306 were enriched in IVBs and of these, 38 were common to both cell types. Figure 3 shows an example of genes that fell into two biological processes (GO Terms) that were common to both cell lines, “response to organic substances” and “regulation of transcription”. These terms were chosen because they had an EASE score < 0.05 and contain nuclear receptors, as well as genes involved in the ETC, CRH regulation, stress responses, and responses to corticosteroid stimuli. Figure 3 also demonstrates that even in biological processes common to both cell lines, the expression pattern is very different between the two. It is interesting to note that with the exception of Hsp90ab1, the AR-5 cells express more heat shock and electron transport genes like DnaJ (Hsp40) homolog subfamily B member 5 (Dnajb5), DNAJ (Hsp40) homolog subfamily C member 3 (Dnajc3) and cytochrome c oxidase subunit Vb (Cox5B), MT-CYB, MT-ND1, MT-ND3. In distinction, the IVB cell line expresses genes involved in corticosteroid metabolism, such as aldehyde dehydrogenase 3 family member A1 (Aldh3a1) and nuclear receptors such as, liver X receptor alpha (Nr1h3), COUP transcription factor 2 (Nr2f2) and the mineralocorticoid receptor (Nr3c2).

Of interest with respect to glucocorticoid down regulation of gene expression is that the activator protein-1 (AP-1) proteins Fos and Jun are differentially expressed between the two lines. *fos*, is expressed approximately 10.2 fold greater in AR-5 s than in IVB, whereas *jun* is expressed approximately 2.2-fold greater in IVB than in AR-5 s (Figure 3). It is known that GRs down-regulate gene expression through AP-1 family members, and Diamond *et al*. showed that the relative abundance of Jun and Fos present with GR at a composite regulatory element determined whether the gene was activated or repressed [15]. Thus, this difference in expression might play a role in one of the differences seen in CRH neurons in the amygdala and the hypothalamus. Specifically, glucocorticoids down-regulate CRH expression in the HPA-axis motor neurons in the PVH, where as they up regulate it in CRH neurons in the amygdala ([16], review).

The Table 2 shows additional biological processes that are of interest, such as, response to hormone stimulus, corticosteroid stimulus, estradiol stimulus, electron transport chain, and cellular response to stress. For each term, the genes identified in Table 2, are a subset of a larger list that can be found in Additional file 1: Table S1. It should also be noted that the genes listed in Table 2 and Figure 3 are not just associated with a single biological process and a complete list of GO Terms associated with genes found in Table 2 can be found in Additional file 2: Table S2. As presented in Table 2, IVB cells express genes involved in responses to hormonal stimuli at a greater level than AR-5, whereas AR-5 cells express respiratory genes and cellular stress response genes at a higher level than the IVB cells.

**Table 2:** **List of select biological processes with an EASE score of** < **0**.**05**, **and their corresponding genes**

**Category**	**Gene symbol**	**Name**	**AR**-**5/IVB**	**EASE score**
Response to hormone stimulus	Adcy5	adenylate cyclase 5	0.394	8.50E-06
	Adcy8	adenylate cyclase 8 (brain)	0.479	
	Adm	Adrenomedullin	0.108	
	Aldh3a1	aldehyde dehydrogenase 3 family, member A1	0.073	
	Arnt2	aryl hydrocarbon receptor nuclear translocator 2	0.223	
	Avpr1a	arginine vasopressin receptor 1A	0.356	
	Eif2b5	eukaryotic translation initiation factor 2B, subunit 5 epsilon	0.409	
	Ghr	growth hormone receptor	0.155	
	Gja1	gap junction protein, alpha 1	0.485	
	Gpx3	glutathione peroxidase 3	0.246	
	Hmgcs2	3-hydroxy-3-methylglutaryl-Coenzyme A synthase 2 (mitochondrial)	0.226	
	Hmox1	heme oxygenase (decycling) 1	0.413	
	Insig2	insulin induced gene 2	0.447	
	Kcnma1	potassium large conductance calcium-activated channel, subfamily M, alpha member 1	0.032	
	Kdm3a	lysine (K)-specific demethylase 3A	0.439	
	lpin1	lipin 1	0.479	
	Nr1h3	nuclear receptor subfamily 1, group H, member 3 (liver X receptor alpha)	0.283	
	Sgk1	serum/glucocorticoid regulated kinase 1	0.293	
	Stat5b	signal transducer and activator of transcription 5B	0.365	
	Stk11	serine/threonine kinase 11	0.471	
	Tgfb3	transforming growth factor, beta 3	0.412	
	Tlr4	toll-like receptor 4	0.138	
Response to corticosteroid stimulus	Adm	Adrenomedullin	0.108	3.90E-02
	Aldh3a1	aldehyde dehydrogenase 3 family, member A1	0.073	
	Avpr1a	arginine vasopressin receptor 1A	0.356	
	Bmp4	bone morphogenetic protein 4	0.276	
	Cav1	caveolin 1, caveolae protein	0.454	
	Ghr	growth hormone receptor	0.155	
	Gpx3	glutathione peroxidase 3	0.246	
	Kcnma1	potassium large conductance calcium-activated channel, subfamily M, alpha member 1	0.032	
	Ptgs1	prostaglandin-endoperoxide synthase 1	0.213	
	Sgk1	serum/glucocorticoid regulated kinase 1	0.293	
	Tlr4	toll-like receptor 4	0.138	
Response to estradiol stimulus	Aldh1a1	aldehyde dehydrogenase 1 family, member A1	0.284	6.10E-03
	Arnt2	aryl hydrocarbon receptor nuclear translocator 2	0.223	
	Bmp4	bone morphogenetic protein 4	0.276	
	CCND2	cyclin D2	0.013	
	Cst3	cystatin C	0.276	
	Gpx4	glutathione peroxidase 4	0.457	
	MAP1B	mi crotubule-associated protein 1B	0.458	
	Pdgfra	platelet derived growth factor receptor, alpha polypeptide	0.071	
	Pdgfrb	platelet derived growth factor receptor, beta polypeptide	0.23	
	Stat5b	signal transducer and activator of transcription 5B	0.365	
Electron transport chain	Cyb561d2	cytochrome b-561 domain containing 2	3.594	1.40E-05
	Cyba	cytochrome b-245, alpha polypeptide	2.186	
	Fads1	fatty acid desaturase 1	2.307	
	GLRX2	glutaredoxin 2	2.158	
	MT-CO1	Cytochrome c oxidase subunit 1	426.042	
	MT-CO2	Cytochrome c oxidase subunit 2	477.423	
	MT-CYB	Cytochrome b	413.38	
	MT-ND1	NADH-ubiquinone oxidoreductase chain 1	874.324	
	MT-ND2	NADH-ubiquinone oxidoreductase chain 2	885.655	
	MT-ND3	NADH-ubiquinone oxidoreductase chain 3	789.947	
	Ndufa10	NADH dehydrogenase (ubiquinone) 1 alpha subcomplex 10	2.972	
	Ndufa11	NADH dehydrogenase (ubiquinone) 1 alpha subcomplex 11	3.222	
	Ndufs1	NADH dehydrogenase (ubiquinone) Fe-S protein 1	2.876	
	Ndufv2	NADH dehydrogenase (ubiquinone) flavoprotein 2	3.599	
	Sod2	superoxide dismutase 2, mitochondrial	5.725	
	Uqcrh	ubiquinol-cytochrome c reductase hinge protein	2.46	
Cellular response to stress	Apoe	apolipoprotein E	2.945	8.20E-04
	ATM	ataxia telangiectasia mutated homolog (human)	2.803	
	Atrx	alpha thalassemia/mental retardation syndrome X-linked (RAD54 homolog, S. cerevisiae)	3.575	
	Bmpr2	bone morphogenetic protein receptor, type II (serine/threonine kinase)	3.772	
	Brca1	breast cancer 1	3.075	
	CCNH	cyclin H	2.232	
	Chek1	CHK1 checkpoint homolog (S. pombe)	2.156	
	Cib1	calcium and integrin binding 1 (calmyrin)	2.176	
	Cryab	crystallin, alpha B	2.567	
	Cxcl10	chemokine (C-X-C motif) ligand 10	3.003	
	Derl2	Der1-like domain family, member 2	2.218	
	DNA2	DNA replication helicase 2 homolog (yeast)	2.158	
	Eif2ak3	eukaryotic translation initiation factor 2 alpha kinase 3	2.058	
	Fads1	fatty acid desaturase 1	2.307	
	Fam175a	family with sequence similarity 175, member A	2.861	
	Fancd2	Fanconi anemia, complementation group D2	2.245	
	Fen1	flap structure-specific endonuclease 1	2.251	
	Fos	FBJ osteosarcoma oncogene	10.247	
	H2afx	H2A histone family, member X	3.259	
	Hdac1	histone deacetylase 1	4.309	
	Hdac6	histone deacetylase 6	3.035	
	HFE	hemochromatosis	6.684	
	Irak1	interleukin-1 receptor-associated kinase 1	2.25	
	Kat5	K(lysine) acetyltransferase 5	2.098	
	Kif22	kinesin family member 22	2.173	
	Nuak2	NUAK family, SNF1-like kinase, 2	2.981	
	Pdcd6ip	programmed cell death 6 interacting protein	2.537	
	Rad18	RAD18 homolog (S. cerevisiae)	2.862	
	Rad50	RAD50 homolog (S. cerevisiae)	2.745	
	Rad51	RAD51 homolog (S. cerevisiae) (RecA homolog, E. coli)	3.009	
	RGD1307983	similar to HSPC043 protein	3.009	
	Rpain	RPA interacting protein	2.024	
	SLK	STE20-like kinase (yeast)	2.698	
	Sod2	superoxide dismutase 2, mitochondrial	5.725	
	Sp100	SP100 nuclear antigen	5.382	
	Stradb	amyotrophic lateral sclerosis 2 (juvenile) chromosome region, candidate 2 (human)	2.154	
	Trip13	thyroid hormone receptor interactor 13	2.417	
	Uhrf1	ubiquitin-like with PHD and ring finger domains 1	2.804	
	Usp1	ubiquitin specific peptidase 1	5.541	
	Xbp1	X-box binding protein 1	3.932	

Genes expressed at ≥ 2 fold are more abundant in AR-5 and genes expressed at ≤ 0.5-fold are highly expressed in IVB cells. A complete list of genes in the above mentioned biological processes can be found in Additional file 1: Table S1 and biological processes associated with a gene can be found in Additional file 2: Table S2.

### Link between CRH and mitochondrial genes

**Figure 4:**
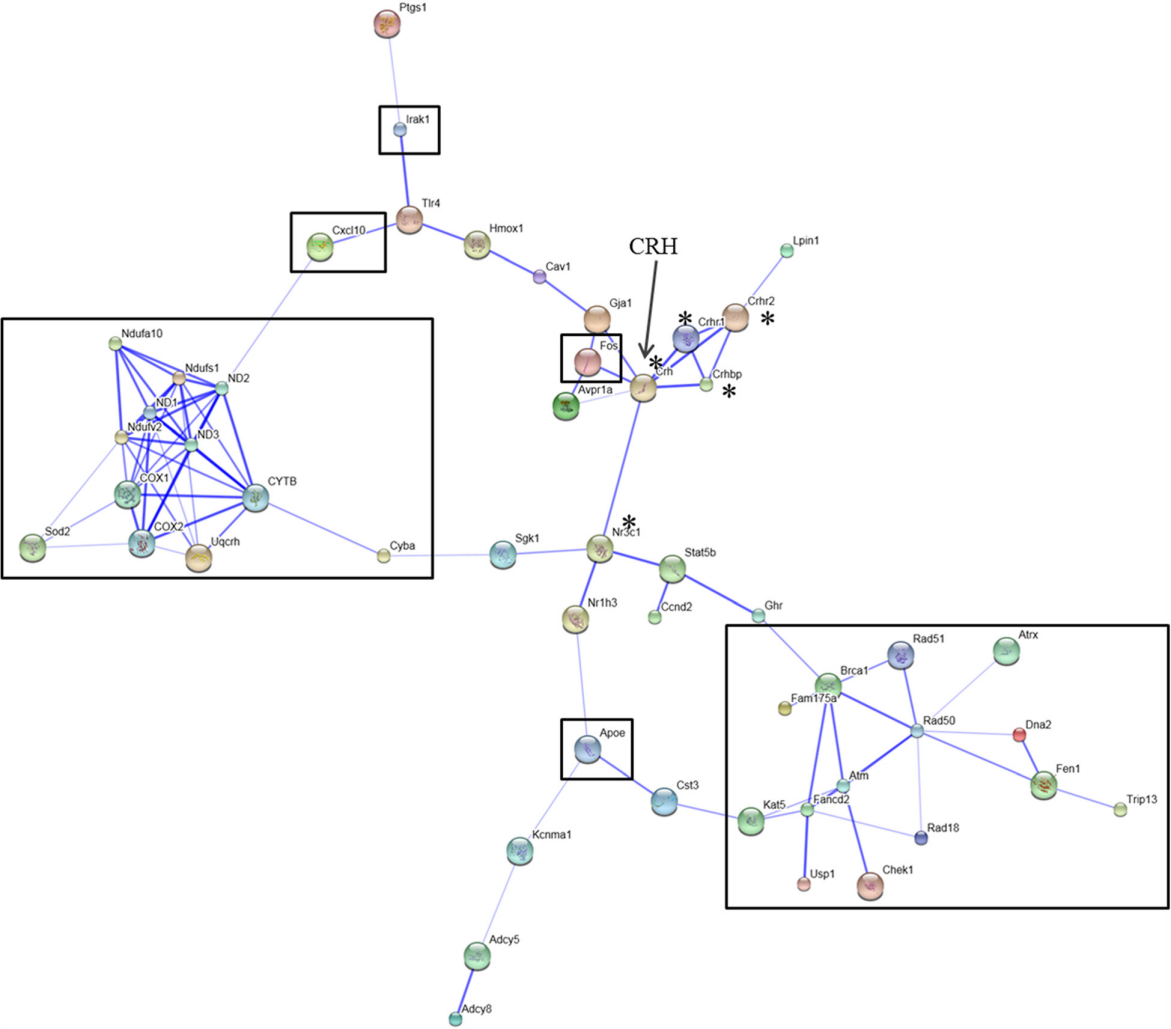
**Interaction between CRH and differentially expressed genes of AR**-**5 and IVB.** Lines connecting each gene indicate either direct physical interaction or indirect functional association. Thicker lines indicate stronger associations. Genes relatively abundant in AR-5 are outlined in black, genes with an asterisk (*) are not differentially expressed, and the remaining genes are relatively abundant in IVBs. See Table 2 for gene names.

In mammals, the endocrine stress response is initiated by activation of the HPA axis, which includes elevated levels of hypothalamic CRH, which ultimately lead to increased levels of circulating glucocorticoids. In the periphery, glucocorticoids stimulate gluconeogenesis and lipolysis to meet increased energy demands associated with a stressful situation. To determine if there is any connection between CRH and the differentially expressed genes between AR-5 and IVB cell lines, genes listed in the Table 2 were submitted to the STRING on-line database analysis tool, which searches for physical and/or functional associations between enriched genes that have been reported in the literature (Figure 4). Figure 4 shows a strong association between the mitochondrial genes (MT-ND1, MT-ND2, MT-ND3, MT-CO1, MT-CO2, MT-CYB, Ndufa10, Ndufs1, Ndufv2), which are abundant in AR-5 cells, and a strong association between genes involved in CRH signaling and regulation (CRH, CRHR1, CRHR2, CRHBP, glucocorticoid receptor (GR or Nr3c1)), which are not differentially expressed. The figure also shows that there is no direct connection between the mitochondrial genes and CRH or GR.

Although STRING did not reveal any direct association between the mitochondrial genes and GR, glucocorticoids have been shown to have a profound impact on mitochondrial functions. They can influence mitochondrial respiration, OXPHOS, ion transport, size and mitochondrial copy number [17-20]. Also, there is evidence to suggest that glucocorticoids modulate mitochondrial transcription through GR activation [21]. The mitochondrial genome contains 6 GRE (glucocorticoid response element)-like sequences, 2 in the displacement loop (D-loop) (GREa, GREb), 3 in the MT-CO1 gene (GRE I-III) and 1 in the MT-CO3 gene (GRE IV) [22]. The mitochondrial genome codes for 13 structural genes, all of which are under the control of a single promoter, the D-loop [23]. GR has been shown to bind to all six GRE sites [21 (Figure 4), 22 (Figure 2)]. GR binding to the two sites in the D-loop may have a direct effect on gene transcription; however, the mechanisms by which GREs I-IV, which are present in the MT-CO1 and 3 coding regions, could regulate gene expression is more enigmatic. The majority of these studies have been done in liver and muscle cells, and we have not come across any studies that examined effects of glucocorticoids on mitochondrial gene expression in the hypothalamus or amygdala.

To begin to determine the effect of glucococrticoids on mitochondrial gene expression, we performed a preliminary experiment in which the cells were treated with 10^-7^ M dexamethasone (Dex, a synthetic glucocorticoid agonist) for 6 hours We measured the change in expression of the six differentially expressed mitochondrial genes identified in the array. Based on a sample size of 2 (n = 2), we observed a Dex-induced increase in expression of 4 (out of 6) genes (MT-CYB, MT-ND1, MT-ND2 and MT-ND3) in the AR-5 cell line and 2 genes (MT-CYB and MT-ND3) in the IVB cell lines (data not shown). Given that we only tested 6 genes and the mitochondrial genome encodes 13 protein coding genes, one of our goals is to study how the remaining mitochondrial genes respond to glucocorticoid treatment. Also, given that all the mitochondrial genes are transcribed as a polycistronic transcript, we expected all 6 genes to respond in the same way. The fact that the genes had different responses, leads us to infer that Dex may be influencing mitochondrial RNA stability as well as mitochondrial transcription. We will pursue these findings in future studies in which we will take microarray, bioinformatic and biochemical approaches to elucidate mechanisms by which glucocorticoids alter mitochondrial gene expression.

## Conclusion

Our goal was to identify genes that were differentially expressed between AR-5 and IVBs. We hypothesized that transcription factors, coregulators and/or chromatin modifying enzymes would be markedly different between the two cell lines. We did find such genes to be differentially expressed but the greatest difference was in six mitochondrial DNA-encoded genes, which are expressed in much greater abundance in the amygdalar AR-5 cell line as compared to the hypothalamic IVB cell line. It is not clear why the expression of mitochondria-encoded genes would be so different between two neuronal cell lines with overlapping functions. One possibility could be that AR-5 cells have a higher energy demand than IVBs. This possibility can be evaluated empirically *in vivo*, as can numerous other questions that will arise from further analysis of these data.

## Abbreviations

CRH: Corticotropin-releasing hormone; MT-CYB: Cytochrome b; MT-CO1: Cytochrome c oxidase subunit 1; MT-CO2: Cytochrome c oxidase subunit 2; MT-ND1: NADH-ubiquinone oxidoreductase chain 1; MT-ND2: NADH-ubiquinone oxidoreductase chain 1; MT-ND3: NADH-ubiquinone oxidoreductase chain 1; OXPHOS: Oxidative phosphorylation; HPA: Hypothalamic-pituitary-adrenal; PVH: Paraventricular nucleus of the hypothalamus; Snx12: Sorting nexin 12; Grem1: Gremlin 1; Actg1: Actin gamma 1; Colec12: Collectin sub-family member 12; Eef2: Eukaryotic translation elongation factor 2; Mmp2: Matrix metallopeptidase 2; Dnajb5: DnaJ (Hsp40) homolog subfamily B member 5; Dnajc3: DNAJ (Hsp40) homolog subfamily C member 3; Hsp90ab1: Heat shock protein 90 kDa alpha class B member 1; Cox5B: Cytochrome c oxidase subunit Vb; Aldh3a1: Aldehyde dehydrogenase 3 family member A1; Nr1h3: liver X receptor alpha; Nr2f2: COUP transcription factor 2; Nr3c1: Glucocorticoid receptor; Nr3c2: Mineralocorticoid receptor; D-loop: Displacement loop; RpL27a: 60S ribosomal protein L27a; GRE: Glucocorticoid response element; NCS: Newborn calf serum; ACTH: Adrenocorticotropic hormone; FDR: False discovery rate; DAVID: Database for annotation visualization and integrated discovery; GO: Gene ontology; EASE: Expression analysis systematic explorer; STRING: Search tool for the retrieval of interacting genes/proteins.

## Competing interests

The authors have nothing to declare.

## Authors’ contributions

DAD and RMU jointly decided to embark on the project. DAD conducted the microarray, RTqPCR, and DAVID analysis and drafted the manuscript. RMU assisted with planning of the project. Both authors edited and approved the final manuscript.

## Supplementary Material

Additional file 1: Table S1 List of select biological processes with an EASE score of < 0.05 and their corresponding genes. Click here for file

Additional file 2: Table S2 Biological processes (BPs) associated with genes identified in Figure 3 and Table 2. Click here for file
